# Mean and volatility spillover in Asian economies: Evidence from trade war

**DOI:** 10.1371/journal.pone.0292819

**Published:** 2023-11-09

**Authors:** Anum Shafique, Nousheen Tariq Bhutta

**Affiliations:** Department of Management Sciences, Capital University of Science and Technology, Islamabad, Pakistan; Vellore Institute of Technology (VIT), INDIA

## Abstract

This study aims to assess the mean and volatility spillover due to trade war between US and China on the Asian markets using GARCH, evidencing that portfolio opportunity exists for the investors in these markets. These markets may offer diversification benefits to investors who fear the negative ramifications of stock markets of the economies in US and China. The study creates a composite variable to test the impact of trade war. The composition of the variable is based on Bilateral Tariffs, Trade policy and Economic policy uncertainty of US only. It means the study covers the US side only for creating a trade war variable. The findings of the study reveal no mean or volatility spillover exists. The study has implications for investors and policymakers.

## Introduction

Financial markets serve as an economic gauge. The increase in market volatility is a major source of concern for policymakers and investors [1]. Because high volatility implies more market risk, it may cause investors to make different decisions. Volatility is threatening the system’s economic viability, according to policymakers. Several studies have been conducted to study the mean and volatility spillover between markets because of various events. The probability of spillover has increased because of global financial market integration. For example, the 2008 financial crisis affected markets worldwide, and a similar situation existed during the period of Covid-19.

The study’s objective is to take into account the trade war issue between US and China and to check its impact on the financial markets. The subject of trade war between US and China has been discussed in the literature for a long time [2] and various reasons are explained that trigger this issue. A few major reasons include protectionism, imposition of trade tariffs [3]. The after effects of this issue are not limited to the two countries (USA or China) only; the ramifications can be seen worldwide. The focus of this study is to check the impact of trade war on selected Asian economies only. The selection of countries for the underlying study is based for the receptiveness of trade war effects by these countries as suggested in the literature.

We use stock market data (Daily average closing price) of four Asian countries: Pakistan, India, Bangladesh and Sri Lanka. Trade War is a composite variable based on three proxies, as suggested by the literature. The data of three proxies (Details of variables and data source are provided in S1 Appendix) is obtained for USA. Yearly and monthly data is converted into daily in EViews software to perform the analysis. The period of study is from January 01, 2014 to December 31, 2022.

Some studies investigated the impact of Trade War on global markets; for instance, [4] studied the impact of trade war on US firms. [5] investigated the impact of Trade War on Chinese markets. Other studies highlight the importance of global financial impact of Trade War [6]. Trade War has a far-reaching impact on the Asian economies, considering the context of globalization and integrated economies. Further, according to a book by Rahul Nath Chaudhry, these countries are highlighted as highly receptive to trade war effects. Moreover, this study also seeks to investigate how Asian stock markets respond to the trade war. If the EMH holds true and the Asian stock market results in no volatility and spillover, it shows that the markets have already discounted the information. No study was found that discusses the impact of trade war by creating a composite variable. The creation of a composite variable gives multidimensional coverage to the variable.

The study’s findings reveal that the market has no means and volatility spillover. Although the Garch Term shows that the volatility and the persistence of volatility exist in the markets, no spillover was found.

### Theoretical background

The realist theory of international trade explains the linkage between the trade war and the stock markets. The theory explains that states become rational utility maximizers in the international system. Their prime purpose is to protect their security and chances of survival. In that context, states may favor trade with political allies instead of adversaries. This may disproportionately benefit the partner countries at their expense [7].

Moreover, investors adjust their portfolios to such economic conditions in order to make portfolio decisions that are better resistant to these circumstances [8]. The stocks of these markets, which have no trade war implications and benefit from trade diversion, may play the role of safe haven for investments. However, a shred of empirical evidence is necessary for investors to rely on this hypothesis. International portfolio theory justifies it.

## Literature review

[9] studied the impact of US-China trade war on the US soybean futures market. [10] highlighted the influence of China on the Asian region is a huge challenge to USA. It can be a game changer geopolitically for the US economy. To counter this challenge, USA keeps on taking different steps.

[3] investigates the impact of the US-China Trade war on the selected economies. The study uses the CGE (Computable General Equilibrium) model of global trade to test this impact. The method of the study was simulation analysis to understand the impact of trade war on tariffs, investment and productivity. The study’s findings reveal that the countries’ gross domestic product (GDP) (US and China) is reduced due to this war. The study also investigates the impact of global value chains to generate trade responses and found these value chains to play a substantial role in creating strong trade responses by the countries under war.

[11] state that physical proximity increases the sensitivity of exposure to the crisis. The firm uses US-China Trade war shocks to test its impact on the firms in China’s stock market to find whether the firms that have spatial proximity have reduced their market value or not due to this event. The findings of the study reveal that the market value of the firms is significantly reduced and there is a spillover effect that can be seen on to the peer organizations.

[12] studied the reaction of exporters because of trade protection due to trade war between US and China. The study’s findings reveal that the exports to China by US decreased and the reduction was made in quantity and not due to change in price. The study also found that the trade shocks due to trade war led the countries to divert exports to other countries.

[13] discuss the causes and the consequences of the trade restricting measures between US and China. The study highlights many causes such as US allegations on China for unfair trade practices, technology theft etc. The study reveals that these allegations turned into battle for leadership for the country. Although the USA has a long technological development history, China now challenges this position.

Lastly, [14] studied the impact of trade war on Asian economies using a neural network approach. The study provides a strong rationale for Asian economies as an effectees of trade war. The study’s findings reveal that there is a need to give importance to the neural network analysis to lessen the impact of trade war.

Keeping all the above study in view, the visibility of Asian stock markets in the context of trade is lacking in the literature. The literature also does not provide any evidence regarding the composite variable to view the issue from a macro perspective. Therefore, this study intends to study the impact of trade war and its mean and volatility spillover on the Asian Markets.

## Methodology

The study considers US data to investigate the impact of trade war on Asian Economies. The reason for selecting the Asian stock markets has been established by two sources first the study conducted by [10] who highlighted the importance of Asian region in the event of US-China trade war. In addition, in a book by Rahul Nath Choudhry, the author clearly stated that South Asian economies are more receptive to trade war. It is to be noted that all the variables to create a composite variable of Trade war are for US. For instance, the EPU and Services Trade Restrictiveness index are taken for the USA, and bilateral Tariffs are tariffs imposed by the USA on Chinese imports. The data for EPU is obtained from policyinsight.com, for TTRI is obtained from OECD. The data for bilateral trade is also available for WITs database. The time period for the study is 2014 to 2022. (A detailed Table for proxy-related information is provided in S1 Appendix).

### Trade war composition

For trade war, a composite variable is created for this study like Economic policy uncertainty (a news-based indicator for USA), Bilateral Tariffs (Imposed by US on Chinese Imports) and Trade Policy (measured through Trade Tariff Restrictiveness index). These proxies were then used to create a composite variable through principal component analysis in EViews software.

### Trade-related proxies

Due to its accuracy in predicting stock market returns, EPU has been utilized widely in the literature. [15] discovered that EPU had a detrimental effect on the US stock market return. [16] explain how the EPU of the US and China affects the bilateral trade balance. According to the analysis, China’s growing unpredictability benefits the US-China trade balance.

[17] found the negative impact of an increase in EPU of US markets on other economies such as China. [18] studied the impact of EPU on the stock market returns during the US-China Trade war and Covid-19 period.

[19] found that the trade policy uncertainty (categorical component of EPU) could be used to predict the US stock returns during the recession period. [20] found that during the period of financial crisis, EPU has shown superior predictive performance. [21] show the impact of EPU on predicting carbon futures. The study’s findings showed that prediction could be made with the help of categorical EPU however, the prediction ability of single categorical EPU is not robust.

Besides EPU index, other proxies, including bilateral tariffs and trade tariff restrictiveness index are also used to measure the impact of trade war in the literature. For instance, [22] used TTRI as it provides a uniform tariff rate that yields the same level of imports as differentiated structure of restrictions. It is accounted for by its narrower trade policy coverage (as it includes only tariffs). As in the case of Bilateral Tariffs, [23] dissected the impact of trade war on US exports by studying the bilateral tariffs. The study’s findings revealed that trade war has amplified repercussions on the US markets as the country has limited ability to direct its exports to the other markets.

### Analysis tool

#### Principal component analysis

The underlying study has used Principal component analysis (PCA) methodology to create a Trade War index to investigate the impact of Trade War on the financial markets. PCA technique in EViews allows the weights to be automatically created for each Trade War component.

### GARCH

The underlying study employs [24] ARMA-GARCH model to calculate the mean and volatility spillover caused by the trade war on financial markets. Two steps are taken to carry out this approach. The return series is modelled in the first stage and mean and return volatility spillover is assessed in the second.

First step:

$$\omega_{k,t}=\tau_{o}+\tau_{1}.\omega_{k,t-1}+\tau_{2}.\vartheta_{k,t}+\tau_{3}.\varepsilon_{k,t-1}+\varepsilon_{k,t},\varepsilon_{k,t}\sim N(0,\vartheta_{k,t})$$

$$\vartheta_{k,t}=\emptyset_{0}+\emptyset_{1}\vartheta_{k,t-1}+\emptyset_{2}\varepsilon_{k,t-1}^{2}$$

Where *ω*_*k*,*t*_ denotes the daily stock market returns at time t and *ε*_*k*,*t*_ denotes the residual or unexpected return. It is also goes by the name “error term”. The ARMA(1,1) GARCH structure is used in the model to correct the data’s autocorrelation.

Standardized residuals are obtained in order to determine the mean return and volatility spillover effects on the financial markets. This equation square from the first stage is then replaced to get the desired outcomes.

Second Step

$$\omega_{j,t}=\tau_{j,o}+\tau_{j,1}.\omega_{j,t-1}+\tau_{j,2}.\vartheta_{j,t}+\tau_{3}.\varepsilon_{j,t-1}+\gamma_{j}\varepsilon_{k,t},\varepsilon_{j,t}\sim N(0,\vartheta_{j,t})$$

$$\vartheta_{j,t}=\emptyset_{j.0}+\emptyset_{j,1}\vartheta_{j,t-1}+\emptyset_{j,2}\varepsilon_{j,t-1}^{2}+\varphi_{j,2}e_{k,t}^{2}$$

Where the trade war’s standardized error term *ε*_*k*,*t*_ captures the effects of the trade war’s mean return and volatility spillover. The square of the standardized error term, which is contained in the conditional volatility equation and has the following definition, is used to investigate the exogenous variable $e_{k,t}^{2}$ and is defined as

$$e_{k,t}=e_{k,t}/\sqrt{\vartheta_{k,t}}$$

## Discussion

Table 1 shows that the lowest value of the standard deviation among the returns of the countries is for Bangladesh (0.07%). The maximum return in a day for the stock market returns is of Bangladesh as well 11.45%. The values for TWR series also returns. This series combines the results of the PCA analysis on the three proxies (Bilateral trade, Trade Policy and EPU). The skewness exhibits asymmetric behavior; it has a positive value for all stock markets other than India and a positive value for the TWR series. Kurtosis explains the data’s shape (or, more specifically, the bell curve’s pointiness). For all of the series, it is also positive and greater than 3, demonstrating that all the series have fat tails and high peaks.

**Table 1:** Descriptive statistics.

	INDR	PAKR	SRIR	TWR	BNR
Mean	-0.000217	-0.000267	-0.000336	-0.004042	0.000108
Median	-0.000236	-0.000345	0.000108	0.000254	9.50E-06
Maximum	0.093477	0.056366	0.092367	1.056775	0.114557
Minimum	-0.160522	-0.078011	-0.08477	-1.354987	-0.059432
Std. Dev.	0.010262	0.00945	0.009603	0.093613	0.007206
Skewness	-1.223952	-0.377418	-0.35849	-1.277913	1.634448
Kurtosis	31.53243	8.82595	16.89389	42.10625	30.76753

Table 2 shows the ARCH effect for all the series. The result in the table shows that the significant value means that the ARCH effect exists in all the series. Therefore, ARMA GARCH model can be applied. If the ARCH value is not significant, other models would be selected and the selection of GARCH model would not have been appropriate.

**Table 2:** Arch effect.

Sr No	Series	LR Statistic	Significance	Model
1	Trade War	609.0321	0.0000	ARMA-GARCH
2	Bangladesh	341.4986	0.0000	ARMA-GARCH
3	Srilanka	243.1203	0.0000	ARMA-GARCH
4	India	237.295	0.0000	ARMA-GARCH
5	Pakistan	93.59945	0.0000	ARMA-GARCH

Table 3 shows the mean and volatility spillover due to the trade war in the Asian Stock markets. The mean returns *γ*_1_ of all the countries show significant results except Bangladesh. It means that only the returns of Bangladesh stock market can be predicted through the pattern of past prices.

**Table 3:** Mean and volatility spillover due to trade war to the Asian stock markets.

	Trade War	P-value	Pak	P-value	India	P-value	Bangladesh	P-value	Srilanka	P-value
*γ*_0_	0.059326	0.0912	0.160801	0.0052	0.124144	0.0267	0.194351	0.0000	0.007889	0.9544
*γ*_1_	0.965383	0.0000	-0.000744	0.1035	-0.000429	0.3337	-0.000694	0.0056	-0.000353	0.8442
*γ*_2_	-0.415531	0.0000	0.45977	0.0000	0.470877	0.0000	0.447167	0.0000	0.888355	0.0000
*γ*_3_			-0.120045	0.0273	-0.156126	0.005	-0.100999	0.0639	-0.298761	0.0000
*ϑ*			-0.000505	0.7814	0.000291	0.8863	0.000283	0.8039	-0.000231	0.9283
*δ*_0_	0.00724	0.0000	3.84E-06	0.0000	2.44E-06	0.0000	1.92E-06	0.0000	5.28E-05	0.0000
*δ*_1_	0.15	0.0013	0.13695	0.0000	0.098314	0.0000	0.190375	0.0000	0.149877	0.0000
*δ*_2_	0.6	0.0000	0.822525	0.0000	0.872357	0.0000	0.780026	0.0000	0.596066	0.0000
*δ*_3_	0	1.0000	1.95E-08	0.9224	1.41E-07	0.4621	2.22E-08	0.7833	-2.02E-06	0.0000

It is also possible today that this market is inefficient. The insignificance of other nation’s stock markets demonstrates the impossibility of predicting present returns from historical prices. For all series exhibiting volatility persistence, the Garch Term *γ*_2_ is important.

The error term *γ*_3_ is also significant for all the series, showing that for the purpose of correction in the future, what will be the direction of the two series and all the statics values (beta values) are negative showing that if the increase, the trade war shall cause the market to move in the opposite direction.

The GARCH term *δ*_1_ is significant for all the series indicating the persistence of volatility. The residual term *δ*_2_ is significant for all the series show that volatility can be forecasted by considering past prices’ behaviour. In order to check the volatility in the long run the summation of *δ*_1_ + *δ*_2_ is obtained. If it is equal to one, it means volatility persists in the long run. This is the case only in the trade war series. The sum is close to 0.96. *δ*_3_ shows the volatility spillover only exists in the Sri Lanka Market whereas mean spillover exists in *γ*_3_ exists in all the markets.

The mean spillover *ϑ* insignificant for all the series. It shows no mean spillover exists from Trade War to the Asian Markets. The volatility spillover *δ*_3_ is significant only for Sri Lanka. It shows Asian markets have no mean and volatility spillover from due to Trade War and offers a diversification benefit for the investors suffering due to trade war in the economies that are parties to the trade war. The findings show harmony with the previous study. [25] state that although the trade war results are negative for both USA and China, other countries experience positive impact due to trade diversion effects. Another study by [26] used to check the spillover effects of China-US trade War on the Southeast Asian Economies. The study’s findings reveal that the third-party country would tend to have the impact of trade war by gaining the spillover effect of the trade war. The research found that these countries are optimal choices for US and China to avoid the tariffs. [27] studied the economic impacts of US-China Trade War on Asian Economies. The findings of the study reveal that some Asian economies take the benefit from this war. The study further states that as the war is bilateral, only the concerned parties are affected by it.

Lastly, [14] used a neural network approach to investigate the impact of the trade war on Asian Economies. The findings of the study reveal that inconsistencies exist in the linkages. It means that there is an impact of trade war on the Asian economies and due attention must be paid to the neural evidence in the policy determination and the trade agreements.

## Conclusion

The focus of the study was to find out the mean and volatility spillover in the Asian economies due to trade war. The findings reveal that either no spillover mean or volatility exists showing a diversification opportunity for the investors in the economies that are parties to the trade war.

This study will help the investor community and policymakers understand the geo-political environment, respond to this type of global issues, and strengthen international trade relations. As [28] found, the result of a trade war brings no winner. Instead, both economies are finding substitutes and thus ultimately benefitting their allies. For instance, many multinational corporations shifted from China to Southeast Asian and other regions in order to avoid spiked tariffs.

A key limitation of the study is included in the scope of this study. It focuses on the South Asian economies, which reduces its generalizability. However, it is insightful if the focus is on the Asian part, thus future research may be done on other blocs that may be affected due to the trade war.

Lastly, this study can be extended in numerous ways. For instance, this composite variable of trade war solely depicted US data. It means Economic policy uncertainty data and trade policy data were of USA. The data of bilateral tariffs was also of those tariffs imposed by US to Chinese imports. Future studies may consider the same from the Chinese Perspective. In addition to this, other regions such as Europe, other economies in Asian region, and other blocks may also be studied as US and China are the world leaders and globalization and financial integrations will cause its systemic impact to spread globally. Finally, other markets, such as currency, cryptocurrency, commodity, etc., may also be explored. More advanced techniques such as VAR-GARCH, BEKK-GARCH, DCC GARCH and Copula methods may also be used.

## Supporting information

S1 Appendix (TIFF)
