# Supplementary figures and images for: Mean and volatility spillover in Asian economies: Evidence from trade war
Source: PLoS One. 2023 Nov 9;18(11):e0292819. doi: 10.1371/journal.pone.0292819 (PMC10635448; doi:10.1371/journal.pone.0292819)

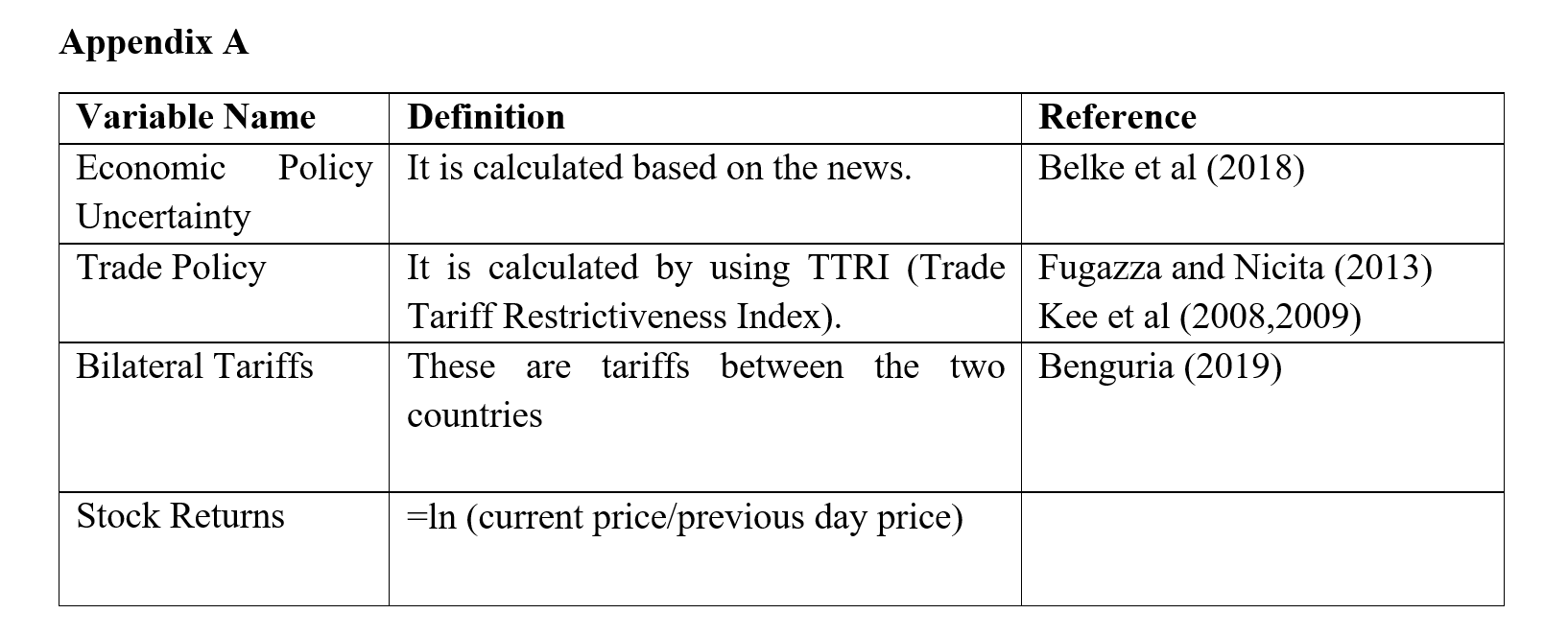

Supplement: S1 Appendix — (TIFF) [file pone.0292819.s001.tiff]
